# Proteome-wide quantification of protein turnover in frog and fly embryos reveals divergent strategies of maternal inheritance

**DOI:** 10.64898/2026.09.28.754234

**Published:** 2026-09-29

**Authors:** Edward R. Cruz, Argit Marishta, Gloria Bao, Alexander N.T. Johnson, Felix C. Keber, Vyas Pujari, Daniil Ivanov, Michael Neinast, Marc W. Kirschner, Joshua D. Rabinowitz, Eric F. Wieschaus, Martin Wuhr

## Abstract

Every embryo inherits a maternal proteome that it must remodel with zygotic proteins to build its many cell types. The fate of the maternal proteome remains contested because indirect measurements cannot resolve it. Here, we combine 18O-water labeling with multiplexed proteomics to quantify protein turnover proteome-wide in frog and fly embryos. Through hatching, the frog preserves the bulk of its maternal proteome, confining rapid degradation to a small regulatory module. The fly cannot meet its synthesis demand from yolk alone and instead degrades nearly all maternal proteins, including housekeeping proteins long assumed stable, recycling them into new protein. Yet the turnover hierarchy is conserved, with disordered and regulatory proteins degrading fastest, while the fly rescales the whole proteome ~eightfold faster. These results recast the developmental proteome as both informational inheritance and metabolic reserve, establish 18O-water labeling as a turnover method for non-feeding organisms, and provide a resource of embryonic half-lives.

## Full Text Availability

The license terms selected by the author(s) for this preprint version do not permit archiving in PMC. The full text is available from the preprint server.

